# Factors affecting purchase intention of organic food products: Evidence from a developing nation context

**DOI:** 10.1002/fsn3.4015

**Published:** 2024-02-16

**Authors:** Marjan Bazhan, Farnam Shafiei Sabet, Nasrin Borumandnia

**Affiliations:** ^1^ Department of Community Nutrition, Faculty of Nutrition Sciences and Food Technology National Nutrition and Food Technology Research Institute, Shahid Beheshti University of Medical Sciences Tehran Iran; ^2^ Department of Food Science and Technology, Faculty of Nutrition Sciences and Food Technology National Nutrition and Food Technology Research Institute, Shahid Beheshti University of Medical Sciences Tehran Iran; ^3^ Urology and Nephrology Research Center Shahid Beheshti University of Medical Sciences Tehran Iran

**Keywords:** attitude, consumers, organic food, purchase intention

## Abstract

The organic food industry has experienced rapid growth in recent decades. The industry is relatively new to developing countries compared to developed countries. Since Iran has the potential to produce organic products, it is essential to study factors that influence consumers' intention to buy them. The present study was designed and implemented for this purpose. In this cross‐sectional study, 520 adults were selected using multistage sampling. Path analysis was employed to test a hypothesized model of predictors of consumer purchase intention for organic food. Attitude, environmental concern, and sensory characteristics directly impacted the intention to purchase organic food (*p* < .01). Knowledge, perceived price, and household size indirectly affected purchase intention through attitude (*p* < .01). Age indirectly affected purchase intention through health consciousness, environmental concern, perceived price, and sensory characteristics (*p* < .01). Subjective norms, health consciousness, and the perceived convenience of purchase had a positive and significant effect on purchase intention, directly and indirectly, through the mediating influence of attitude (*p* = .000). The educational level also affects purchase intention directly and indirectly through knowledge, health consciousness, environmental concern, and subjective norm (*p* = .000). The most potent total effects belonged to health consciousness, subjective norms, and education, respectively. Overall, the model could explain 47% of the intention variance and 45% of the variance in attitude. Various factors influence Iranian consumers' decision to buy organic food. This information can help professionals make well‐informed decisions in policymaking, production, marketing, tourism, and retailing.

## Graphical abstract

The present study proposed a conceptual model to identify the factors determining the consumer's attitude and intention to purchase organic food. The results showed that attitude, environmental concern, and sensory characteristics had a direct effect on the intention to purchase organic food. The impact of knowledge, perceived price, age, and household size was only indirect. Subjective norms, health consciousness, perceived convenience of purchase, and education level impacted the intention to purchase these products, both directly and indirectly through attitude.

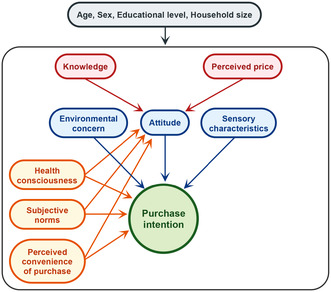

## 1 INTRODUCTION

Environmental problems, such as soil erosion, water pollution from hazardous chemicals like nitrates and pesticides, and the release of greenhouse gases, have extensive implications for public health. Increasing environmental concerns and the desire for chemical‐free food have caused demand for sustainable farming methods, specifically organic farming (Yu & Wu, 2018). The benefits and drawbacks of organic foods have been widely debated since their introduction. Proponents of these products believe that organic farming aims to increase productivity in the long term. According to them, using fewer chemical fertilizers and synthetic pesticides in organic agriculture can enhance biodiversity and soil quality while decreasing ecological concerns (Clark, 2020; Smith et al., 2019). They also emphasize that organic products contain lower amounts of pesticides, leading to fewer health risks (Huber et al., 2012; Rembiałkowska, 2007). Recently, proponents of organic farming have argued that this type of farming might serve as a strategy for adapting to climate change (Guzmán & Alonso, 2008; Scialabba & Müller‐Lindenlauf, 2010).

Organic farming is a rapidly growing industry worldwide. According to the 2023 World of Organic Agriculture Statistics (Willer et al., 2023), Oceania has the most extensive organic agricultural area, with 36.0 million hectares, followed by Europe with 17.8 million hectares, representing 47% and 23% of the world's organic agricultural area, respectively. In Asia, 6.5 million hectares are managed under organic agriculture, representing almost 9% of the world's organic agricultural land. However, in developing countries such as Iran, there appears to be no discernible effort to plan, guide, or support organic agriculture. Despite having great potential for organic farming due to its water and soil resources, climate, and genetic diversity (Khosh‐Khui, 2016), Iran has not yet taken advantage of this opportunity. Population growth and the decision of policymakers to produce as much as possible to achieve food security and to sacrifice quality for quantity are some of the primary reasons for the neglect of organic agriculture in Iran. The reluctance of farmers to accept organic production due to fears of a possible reduction in yield per unit area and, consequently, a decrease in income can be another obstacle (Hosseini, 2019). According to the Global Report 2018, about 12,000 hectares of agricultural land in Iran are allocated to cultivate organic products. Iran's share in the worldwide cultivation of organic products was about 0.04% in 2018 (Willer & Sahota, 2020). Meanwhile, in the Fifth Development Plan (2013–2017), it was predicted that about 25% of agricultural lands and gardens (equivalent to 4 million hectares) should be covered by the production of organic products. Surveys have shown that 300 to 400 thousand hectares of agricultural land should be under organic cultivation to achieve an optimal supply situation in the country (Hosseini, 2019).

Iranian consumers are increasingly interested in food safety, quality, longevity, and improving personal and social well‐being. This demand, combined with Iran's diverse climate and abundant natural resources, makes it conducive to producing organic products. It is important to note that consumer demand is a significant and influential factor in producing organic agricultural produce. Therefore, it is essential to investigate the factors that affect consumers' willingness to purchase and consume such products. A comprehension of consumer behavior aids in the development of improved marketing strategies and production processes (Yeganeh et al., 2021).

The purchasing behavior of consumers toward organic food has mainly been analyzed in developed countries. In Iran, a developing country, limited research has been conducted on this issue in some cities (Bagher et al., 2018; Imani et al., 2021; Yazdanpanah et al., 2015; Yazdanpanah & Forouzani, 2015). Identifying the local factors that impact the consumption of organic foods is essential for effective future planning, as these factors can vary significantly across various sociocultural contexts. Therefore, the present study was designed and implemented for this purpose in Tehran, Iran's capital city and one of its most prosperous cities, regarding economic and social indicators. The theory of planned behavior (TPB) was used as a conceptual framework to understand consumers' purchasing intentions toward organic foods. TPB is a highly influential social and health psychology theory, highlighting the complex relationship between human behavior and its determinants. This framework emphasizes that human behavior results from a deliberate intention to execute a specific action (Onwezen et al., 2013). The study has extended the TPB framework by adding some independent variables that have been suggested to play an essential role in determining consumer behavior toward sustainable products and, in particular, toward organic food: knowledge, health consciousness, environmental concern, perceived price, perceived convenience of purchase, sensory characteristics, and sociodemographic characteristics, and explored how these factors influence consumers' intention to purchase organic food. Further details on these inclusions are provided in the following section. To the best of the authors' knowledge, few studies have investigated the factors influencing the intention to purchase organic food using TPB in the Iranian context. This study aimed to examine the effect of different factors on the intention to buy organic food and test the mediating role of attitude between the examined factors and the intention to buy organic food.

## 2 THEORETICAL FRAMEWORK AND THE DEVELOPMENT OF HYPOTHESES

The TPB was proposed by Icek Ajzen (1991). This framework states that attitude, subjective norms, and perceived behavioral control are predictors of behavioral intention (Ajzen, 1991). The TPB is useful in predicting consumer intention in various contexts (Yang et al., 2018), including organic foods (Rana & Paul, 2017). Indeed, previous studies aimed at investigating and explaining consumers' intentions to purchase organic food have successfully used this model (Ahmed et al., 2021; Boobalan et al., 2021; Nguyen et al., 2021; Tran & Nguyen, 2021; Yang et al., 2018). In addition, it has been reported that the TPB is suitable for predicting the intention to buy organic food in different cultures. However, the variables' relative influence varies from country to country (Yadav & Pathak, 2016).

Although the success of the TPB in predicting behavioral intention and behavior has been demonstrated, the evolution of the theory has not stopped, as Ajzen (1991) suggested it can be deepened and extended by adding new elements or redirecting existing ones. Scientists believe that including more variables in TPB can enhance its predictability (Arvola et al., 2008; Donald et al., 2014). So, the present study has expanded the TPB framework by incorporating additional structures, drawing on existing literature, to enhance our understanding of consumers' behavioral intentions toward organic foods. It is important to note that in the present study, the conceptual model included perceived price and perceived convenience of purchase instead of perceived behavioral control. Perceived behavioral control refers to the ease or difficulty of performing a behavior, reflecting experience and anticipated obstacles (Ajzen, 1991). It depends on perceived barriers and ability and, in turn, affects consumer purchase intentions (Thøgersen, 2016). Price and availability have been identified as the main barriers to purchasing organic food (Magnusson et al., 2001). Therefore, this study investigated the impact of these two variables on attitude and purchase intention. Based on that, the study hypotheses were formed.

### 2.1 Attitude toward organic foods

Attitude is a disposition to respond favorably or unfavorably to something (Fishbein & Ajzen, 2011). It refers to one's negative or positive assessment of performing a particular behavior. Some researchers have reported that consumers' attitudes toward organic food predict their buying intention (Chu, 2018; Eberle et al., 2022; Parashar et al., 2023). In consequence, the study's first hypothesis is as follows:

Hypothesis 1 Attitude toward organic foods positively affects consumers' intention to purchase these products.

### 2.2 Knowledge about organic foods

Consumer knowledge affects how information is searched, processed, and decided (Stanton & Cook, 2019). Knowledge can also affect trust in a novel product in the market. In contrast, the absence of novel knowledge could diminish consumers' confidence in the information they get (Hossain & Lim, 2016). Aertsens et al. (2011) reported that most individuals consume organic foods; however, their insufficient knowledge makes it hard to differentiate between organic and non‐organic foods. In a recent study in Malaysia (Hossain & Lim, 2016), knowledge was cited as one factor influencing consumers' intentions to buy organic foods.

Sharing more information about organic food could enhance consumer attitudes toward it (Andervazh et al., 2020; Singh & Verma, 2017; Smith & Paladino, 2010) and raise their propensity to pay a premium price (Barnes et al., 2009). Padel and Foster (2005) suggest that consumers will be more inclined to purchase organic foods if they are aware of the reasons for the increased prices. The following hypotheses can be proposed based on this discussion:

Hypothesis 2 Knowledge about organic food positively affects (a) the attitude toward organic food and (b) the intention of consumers to purchase these products.

### 2.3 Subjective norm

A subjective norm is the perceived social pressure to engage or abstain from a particular behavior (Ajzen, 1991). Studies concerning organic foods have shown different relationships between subjective norms and attitudes. According to some researchers, subjective norms do not affect the consumers' intention to purchase organic foods (Yadav & Pathak, 2016; Zayed et al., 2022). However, other researchers found a significant link between the subjective norm and the intention to purchase these products (Carfora et al., 2019; Liobikienė et al., 2016). Instead, Tarkiainen and Sundqvist (2005) found that subjective norms, mediated by attitude, affect the intention to buy organic foods. Irianto's study (Irianto, 2015) showed the direct and indirect effects of a subjective norm (mediated by attitude) on the intention to purchase these products. Contradictions lead researchers to investigate the impact of subjective norms on attitudes and intentions toward purchasing organic food. The following hypothesis is suggested based on this:

Hypothesis 3 Subjective norm positively affects (a) the attitude toward organic food and (b) the intention of consumers to purchase these products.

### 2.4 Health consciousness

Health consciousness is characterized by the inclination toward prioritizing one's health (Iversen & Kraft, 2006). Paul and Rana (2012) reported that people had more positive attitudes toward buying organic foods when they were concerned about their health. Consumers believe that organic foods grow naturally; hence, they are deemed healthier and safer than conventional foods (Doni et al., 2018). According to Loebnitz and Aschemann‐Witzel (2016), perceived health‐related variables help explain the selection of organic foods.

Various cross‐sectional studies have shown that health is integral to organic food consumption (Chakrabarti, 2010; Chen, 2009). Findings from qualitative studies are in the same direction (Padel & Foster, 2005; Zanoli & Naspetti, 2002). The discussion leads to the proposal of the following hypothesis:

Hypothesis 4 Health consciousness positively affects (a) the attitude toward organic food and (b) the intention of consumers to purchase these products.

### 2.5 Environmental concern

The degree to which individuals are conscious of and endorse initiatives to resolve environmental issues is called environmental concern (Dunlap & Jones, 2002). This issue is essential in determining the intention to purchase organic foods (Smith & Paladino, 2010). According to Squires et al. (2001), people who prefer organic foods are more disposed toward being environmentally conscious. Stated differently, the greater the environmental concerns, the more organic foods are consumed. Following the discussion, the following hypothesis has been planned:

Hypothesis 5 Environmental concern positively affects (a) the attitude toward organic food and (b) the intention of consumers to purchase these products.

### 2.6 Perceived convenience of purchase

Convenience is a factor that affects consumer choice behavior. Occasional consumers of organic foods and those who do not use these products pay close attention to convenience issues as a criterion for purchase (Fotopoulos & Krystallis, 2002; Zanoli & Naspetti, 2002). Valuing convenience in food choices negatively affects attitudes toward organic foods (Janssen, 2018) and buying behavior (Moser, 2016). The lack of organic food availability raises search expenses, resulting in a negative convenience effect on food purchases (Li et al., 2007). Other aspects of convenience, such as product placement in the store (Castro et al., 2018) and packaging features (Vilnai‐Yavetz & Koren, 2013), can affect consumers' buying intentions. As a result, the following hypothesis is proposed:

Hypothesis 6 Perceived convenience of purchase positively affects (a) the attitude toward organic food and (b) the intention of consumers to purchase these products.

### 2.7 Perceived price

Based on the traditional economic theory, the price of a product is considered the financial sacrifice required to purchase it. Higher prices negatively affect product evaluation and purchase intention (Sadiq et al., 2020). Price seems dominant in buying organic foods, probably because of the belief that these products are expensive (Padel & Foster, 2005). As a result, high prices are one of the main obstacles to buying organic foods (Aertsens et al., 2009; Nguyen et al., 2019). Hence, the more consumers perceive the price of organic foods to be high, the less favorable their attitude toward purchasing them. This discussion leads to the proposal of the following hypothesis:

Hypothesis 7 Perceived price negatively affects (a) the attitude toward organic food and (b) the intention of consumers to purchase these products.

### 2.8 Sensory characteristics

Sensory characteristics related to food's appearance, smell, and taste have long been known as one of the primary determinants of food choice (Steptoe et al., 1995). Some researchers have observed that sensory characteristics are an essential motivator for buying organic foods (Magnusson et al., 2001; Massey et al., 2018). The sensory properties of organic foods stimulate consumers' emotional/experiential effects; therefore, their evaluation will primarily be based on the hedonic value (Lee & Yun, 2015). Some studies suggest that the sensory characteristics of organic food, such as taste, color, and texture, can evoke feelings of happiness and pleasure (Padel & Foster, 2005; Zanoli & Naspetti, 2002). Thus, the subsequent hypothesis is proposed:

Hypothesis 8 Sensory characteristics positively affect (a) the attitude toward organic food and (b) the intention of consumers to purchase these products.

### 2.9 Sociodemographic characteristics

Studies have shown that social and demographic factors can impact organic food consumption, although the results are inconsistent. Some studies (Aertsens et al., 2009; Paul & Rana, 2012; Sangkumchaliang & Huang, 2012) have reported that women are more favorable toward organic foods than men. A study in Thailand found that the age range of consumers of organic foods in Bangkok is higher than other consumer groups (Roitner‐Schobesberger et al., 2008). Also, studies in Spain (Gutiérrez‐Villar et al., 2022) and Switzerland (Siegrist & Hartmann, 2019) showed that older consumers are more inclined to consume organic foods than younger consumers. Studies in the United States (Durham & Andrade, 2005), the United Kingdom (Rimal et al., 2005), and Sweden (Magnusson et al., 2001) have reported conflicting results. However, Lockie et al. (2004) did not observe a difference in organic food consumption between different age groups. In terms of income, a study by Rimal et al. (2005) found that household income positively affected the likelihood of buying organic food in the United Kingdom. Also, some studies have reported that wealthy households are more likely to pay for organic food (Timu et al., 2022). In addition, it is interesting to note the results of a study by Byrne et al. (1991), which showed an inverse relationship between income and food safety concerns. This finding suggests that wealthy consumers may feel more confident in food safety or be less concerned about pesticide residues because of higher prices (Govindasamy & Italia, 1999).

Concerning education, some researchers found that people with higher levels of education were more inclined to buy organic food than others (Gundala & Singh, 2021; Siegrist & Hartmann, 2019). In contrast, a negative relationship between these two variables was reported in another study (Durham & Andrade, 2005; Magnusson et al., 2001). Some studies have shown no impact of education on organic food purchase patterns (Tran & Nguyen, 2021). However, Li et al. (2007) argued that demographic variables cannot predict organic food buying behavior well. Instead, behavioral and attitudinal variables are better predictors. This research examined how age, gender, educational attainment, and household size impacted the listed variables. So, the following hypothesis is proposed:

Hypothesis 9 Sociodemographic characteristics have a positive impact on all variables listed above.

**FIGURE 1:**
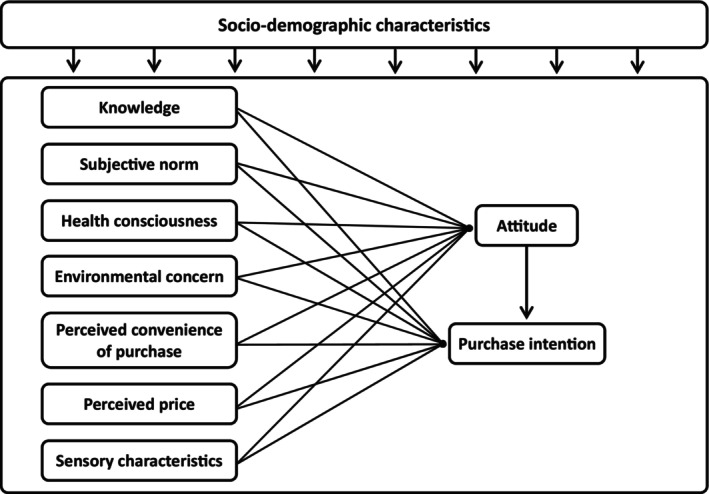
Proposed theoretical framework.

Based on the above discussion, a research framework has been developed (Figure 1 shows the theoretical framework used in the study).

## 3 METHODS

### 3.1 Research design, setting, and subject of study

This cross‐sectional survey was conducted using a multistage random cluster sampling design from June to September 2019. The target population in this study was adults aged 25 to 65 years who went to wholesale fruit and vegetable markets in Tehran, the capital of Iran. According to the number of questionnaire items (52 items) and considering 10 subjects for each item (Hooman, 2016), the sample included 520 people. The criteria used to select the sample were familiarity with organic food, residence in Tehran, age between 25 and 65, and willingness to participate. First, Tehran was divided into five geographical regions: North, South, Centre, West, and East, to ensure representation of all socioeconomic groups. Tehran is somewhat stratified by geographical location, with the south traditionally consisting of a population of lower socioeconomic status. Five districts were then randomly selected, one from each region. The sample was distributed proportionally across the areas according to the population size of each region. Two wholesale fruit and vegetable markets were randomly selected from each district using a list of all such markets (a total of 10 markets). Samples were selected from the markets using inclusion criteria. They were provided with an informed consent form and a research questionnaire. They were assured of confidentiality during the consent process and could withdraw from the study or skip any questions they did not wish to answer. Participants' names were not recorded to ensure accurate responses. The questionnaire took approximately 20–25 min to complete.

### 3.2 Data collection

#### 3.2.1 Assessing the determinants of the intention to purchase organic food

We used a self‐administered questionnaire to study the factors influencing the intention to buy organic foods. Details of developing and validating this questionnaire are explained elsewhere (Bazhan et al., 2023). The questionnaire comprised 52 items, which were categorized into nine distinct sections, namely knowledge (10), subjective norms (3), attitude (7), environmental concern (9), health consciousness (8), perceived price (5), perceived convenience of purchase (4), sensory characteristics (4), and intention to buy organic food (2). Consumer knowledge was evaluated based on three options: correct, wrong, and I do not know. Each correctly answered item scored 1 point. A Likert‐type scale with 5 points was used to rate the other questionnaire items that ranged from 0 (completely disagree) to 4 (completely agree). A reverse score was given to items with negative expressions.

#### 3.2.2 Assessing sociodemographic characteristics

Sociodemographic indicators were collected using a self‐administered questionnaire. The information included personal and household details like age, gender, marital status, education, job status, household size, presence of children below 18 years old, responsibility for household food shopping, and geographical area of residence.

### 3.3 Statistical analysis

The data analysis was conducted using SPSS‐V26 and AMOS‐V26 software. Descriptive analyses were used to describe the subjects' characteristics. Skewness and kurtosis were checked to examine the normal distribution of the data. Path analysis was employed to test and modify the model. Pearson correlation tests were run to check for path analysis requirements. Statistical significance was defined as *p* < 0.05. The model fit was assessed using multiple indices, such as Chi‐square fit index (χ^2^/df), the root mean square error of approximation (RMSEA), normed fit index (NFI), non‐normed fit index (NNFI), incremental fit index (IFI), comparative fit index (CFI), goodness‐of‐fit index (GFI), and adjusted goodness‐of‐fit index (AGFI). A χ^2^/df value less than 2.0 shows an excellent model fit, a value between 2.0 and 5.0 shows a good model fit, and a value greater than 5.0 represents a poor and unacceptable model fit. The RMSEA should be less than 0.05, meaning a good fit. Concerning GFI, AGFI, CFI, IFI, NFI, and NNFI, values no less than 0.90 show well‐fitting models (Tabachnick et al., 2007).

The significance of direct and indirect variable effects was examined using the bootstrap resampling method. The process should be repeated multiple times (typically 1000) during analysis to generate a more reliable confidence interval (MacKinnon et al., 2004). A 95% confidence interval was obtained through 5000 times bootstrapping in this study. The squared multiple regression correlation coefficients (*R* ^2^) were used to determine how much the suggested factors accounted for the variations in purchase intentions.

## 4 RESULTS

### 4.1 Characteristics of the sample

The mean age of the subjects was 37.8 ± 10.7 years, with a considerable percentage being in the age range of 25–34 years. Slightly more than half of the population was women. The majority held an upper diploma degree. Most of the participants were married with a household size of 3–4 individuals, employed, and responsible for household food shopping. Less than half of them had children under 18 in their households (Table 1).

**TABLE 1:** Sample characteristics (*N* = 520).

Characteristic	*n* (%)
Gender	
Female	296 (56.9)
Male	224 (43.1)
Age (year)	
25–34	237 (45.6)
35–44	141 (27.1)
45–54	97 (18.7)
55–65	45 (8.7)
Marital status	
Married	329 (63.2)
Unmarried//Divorced/Widow	191 (36.8)
Household size	
<3	100 (19.2)
3–4	344 (66.2)
5^+^	76 (14.6)
Education	
Under diploma	37 (7.1)
Diploma	121 (23.3)
University	362 (69.6)
Employment status	
Unemployed	92 (17.7)
Worker	10 (1.9)
Employee	192 (36.9)
Self‐employed	103 (19.8)
Retired	17 (3.3)
Housewife	106 (20.4)
Children ≤ 18 years	
Yes	215 (41.3)
No	305 (58.7)
Responsible for household food shopping	
Yes, always	261 (50.2)
No	259 (49.8)
Residential area	
North	103 (19.9)
South	99 (19.1)
Center	110 (21.2)
West	98 (18.9)
East	110 (19.9)

### 4.2 Modification of the original model

Pearson correlation was first used to analyze the bivariate relationships between the variables (Table 2). Attitude had a positive correlation with all variables except for perceived price. Purchase intention was positively associated with all dimensions except the perceived price. The proposed associations between purchase intention and perceived price did not reach statistical significance (*r* = .015, *p* = .746). Consequently, this pathway was removed from the model. Following this, path analysis was performed to assess and amend the proposed research model. However, data normality was examined using skewness and kurtosis before applying the measurement model. The data exhibited normal distribution, as evidenced by skewness and kurtosis values below ±3 and ± 10, respectively. The critical ratio skewness and kurtosis values did not exceed the absolute value of 2.58 (Kline, 2011).

**TABLE 2:** Correlation matrix of study variables (*N* = 520).

Variable	1	2	3	4	5	6	7	8	9	10	11	12
1. Knowledge	1											
2. Attitude	.476**	1										
3. Mental norm	.362**	.527**	1									
4. Health consciousness	.488**	.557**	.491**	1								
5. Environmental concerns	.406**	.426**	.405**	.610**	1							
6. Perceived ease of purchase	.061	.160**	.130**	.096*	.001	1						
7. Perceived price	−.121**	−.217**	−.149**	−.066	.055	.080	1					
8. Sensory characteristics	.328**	.205**	.245**	.304**	.281**	.086	.141**	1				
9. Intention to buy	.370**	.495**	.522**	.561**	.485**	.212**	.015	.366**	1			
10. Age	−.060	−.031	−.118**	.131**	.038	−.030	.092*	.090*	−.002	1		
11. Education	.157**	.160**	.117**	.124**	.167**	.057	−.002	.013	.202	−0.318**	1	
12. Household size	.079	.149**	.019	.069	.122**	.013	.009	−.011	.089*	−.145**	.688**	1

* *p* < .05

** *p* < .01.

The present study's conceptual model showed a poor fit (χ^2^/df = 43.76, GFI = 0.72, AGFI = 0.09, IFI = 0.42, CFI = 0.40, NFI = 0.41, and RMSEA = 0.29). Four pathways were removed based on theoretical reasoning and modification indices to create a parsimonious model. The pathways were knowledge and purchase intention, perceived price and purchase intention, sensory characteristics and attitude, and environmental concerns and attitudes. To achieve the best‐fit model, we conducted multiple tests to compare the modified models (χ^2^/df = 1.69, GFI = 0.98, AGFI = 0.96, IFI = 0.99, CFI = 0.99, NFI = 0.97, and RMSEA = 0.037).

### 4.3 Hypotheses testing

Table 3 outlines the hypothesis testing. Most hypotheses were accepted except for a few hypotheses. H2b and H7b were not supported because knowledge and the perceived price did not significantly affect the purchase intention. Environmental concerns and sensory characteristics did not significantly influence the attitude; thus, H5a and H8a were unsupported. Some segments of H9 were also not supported, as evidenced below. Age did not significantly affect the knowledge, attitude, subjective norm, perceived convenience of purchase, and purchase intention. A similar outcome was achieved regarding the influence of education on attitude, sensory characteristics, perceived price, and perceived convenience of purchase. The impact of household size on the study variables, except for attitude, was insignificant.

**TABLE 3:** Results of the hypotheses tests.

Hypothesized path	*β* value	*t*‐value	*p*‐value	Result	Hypothesized path	*β* value	*t*‐value	*p*‐value	Result
H1: AT➔PI	.124	2.940	.003	Supported	H9‐1g: A➔PP	.084	2.008	.045	Supported
H2a: K➔AT	.202	5.270	.000	Supported	H9‐1h: A➔SC	.106	2.558	.011	Supported
H2b: K➔PI	−.016	−.432	.666	Not supported	H9‐1i: A➔PI	.017	.458	.647	Not supported
H3a: SN➔AT	.273	7.020	.000	Supported	H9‐2a: EL➔K	.142	3.403	.000	Supported
H3b: SN➔PI	.223	5.467	.000	Supported	H9‐2b: EL➔AT	−.052	−1.276	.066	Not supported
H4a: HC➔AT	.306	7.367	.000	Supported	H9‐2c: EL➔ SN	.108	2.514	.012	Supported
H4b: HC➔PI	.224	4.709	.000	Supported	H9‐2d: EL➔HC	.181	4.208	.000	Supported
H5a: EC➔AT	.082	1.885	.061	Not supported	H9‐2e: EL➔EC	.192	4.372	.000	Supported
H5b: EC➔PI	.148	3.462	.001	Supported	H9‐2f: EL➔PCP	.087	1.950	.064	Not supported
H6a: PCP➔AT	.095	2.865	.004	Supported	H9‐2g: EL➔PP	.026	.576	.565	Not supported
H6b: PCP➔PI	.124	3.749	.000	Supported	H9‐2h: EL➔SC	.084	1.897	.068	Not supported
H7a: PP➔AT	−.143	−4.284	.000	Supported	H9‐2i: EL➔PI	.160	4.168	.000	Supported
H7b: PP➔PI	.059	1.628	.104	Not supported	H9‐3a: HS➔K	−.054	−1.233	.217	Not supported
H8a: SC➔AT	−.014	−.379	.705	Not supported	H9‐3b: HS➔AT	.108	3.247	.001	Supported
H8b: SC➔PI	.170	4.834	.000	Supported	H9‐3c: EL➔SN	−.086	−1.893	.062	Not supported
H9‐1a: A➔K	−.006	−.142	.887	Not Supported	H9‐3d: EL➔HC	−.057	−1.331	.183	Not supported
H9‐1b: A➔AT	−.005	−.116	.907	Not Supported	H9‐3e: EL➔EC	−.001	−.014	.989	Not supported
H9‐1c: A➔SN	−.080	−1.829	.067	Not Supported	H9‐3f: EL➔PCP	−.048	−1.078	.281	Not supported
H9‐1d: A➔HC	.224	6.139	.000	Supported	H9‐3g: EL➔PP	.006	.136	.892	Not supported
H9‐1e: A➔EC	.129	3.193	.001	Supported	H9‐3h: EL➔SC	−.053	−1.201	.230	Not supported
H9‐1f: A➔PCP	−.009	−.204	.839	Not Supported	H9‐3i: HS➔PI	−.071	−1.374	.169	Not supported

Abbreviations: A, age; AT, attitude; EC, environmental concerns; EL, educational level; HC, health consciousness; HS, household size; K, knowledge, SN, subjective norm; PCP, perceived convenience of purchase; PI, purchase intention; PP, perceived price; SC, sensory characteristics.

### 4.4 Predictors of buying intentional organic food products in Iranian consumers

Table 4 summarizes each variable's standardized direct, indirect, and total effects on purchase intention. We applied a bootstrapping test to understand the mediation effects. We drew 5000 bootstrapping samples and computed 95% confidence intervals. Attitude, environmental concern, and sensory characteristics only directly affected the intention to buy organic food (*p* < .01). Factors like knowledge, perceived price, and household size indirectly impacted the purchase intention through attitude (*p* < .01). Age had an indirect effect on the intention to purchase organic food, mediated by health consciousness (0.224), environmental concern (0.129), perceived price (0.084), and sensory characteristics (0.106) (*p* < .01). Additional variables, such as subjective norms, health consciousness, and perceived purchase convenience, notably impacted the intention to purchase organic food, both directly and indirectly through attitude (*p* = .000). The education level had a significant impact on the intention to purchase organic food, both directly and indirectly, through its effects on knowledge (0.142), health consciousness (0.181), environmental concern (0.192), and subjective norms (0.108) (*p* = .000). Except for the perceived price, all variables have positively impacted the intention to purchase organic food.

**TABLE 4:** Standardized direct, indirect, and total effects of variables on purchase intentions of organic food (*N* = 520).

Variables	Standardized		Standardized		Standardized	
	Direct effect		Indirect effect		Total effect	
	*β*‐value	*p*‐value	*β*‐value	*p*‐value	*β*‐value	*p*‐value
Knowledge	‐	‐	.025	.004	.025	.004
Attitude	.124	.003	‐	‐	.124	.006
Subjective norm	.223	.000	.034	.004	.257	.000
Health consciousness	.224	.000	.038	.004	.262	.000
Environmental concerns	.148	.001	‐	‐	.148	.001
Perceived ease of purchase	.124	.000	.012	.005	.136	.000
Perceived price	‐	‐	−.018	.003	−.018	.003
Sensory characteristics	.170	.000	‐	‐	.170	.000
Age	‐	‐	.094	.000	.096	.000
Education	.097	.005	.107	.000	.204	.000
Household size	‐	‐	.013	.005	.013	.005

Among organic food purchase intention, predictors, health consciousness, and subjective norms had the greatest total effect, while household size and the perceived price had the lowest total effect (Table 4).

The final model accounted for 45% and 47% of the variance in attitude and purchase intention of organic food, respectively.

To analyze the mediating role of sex in the overall model, we performed two independent estimations by AMOS software: once for 296 women and another time for 224 men. The aim was to compare the hypotheses in these two scenarios. Most relationships in the general model for women were upheld, except those involving environmental concern and purchase intention, education level and knowledge, and household size and attitude. It means that hypothesis H5b and two relationships related to hypothesis H9 were not supported among women. The final model failed to support the relationships between the perceived convenience of purchase and attitude; attitude and purchase intention; age with sensory characteristics, environmental concern, and perceived price; and education level with the subjective norms and purchase intention among men. This group did not support hypotheses H6a and H1 and five relationships related to hypothesis H9.

## 5 DISCUSSION

The current study used TPB and made further efforts to incorporate important constructs such as knowledge, health consciousness, environmental concern, perceived price, perceived convenience of purchase, and sensory characteristics into the TPB model to understand consumers' intention to purchase organic food. The effectiveness of these factors was subsequently evaluated. The results showed that attitude directly and significantly affected the intention to purchase organic food. Previous research has reported that a positive attitude toward organic food leads to an increased willingness to buy them (Carfora et al., 2019; Wang et al., 2019; Yadav & Pathak, 2016). According to our findings, knowledge indirectly impacted the intention to buy organic food through its influence on attitude. Earlier studies (Andervazh et al., 2020; Singh & Verma, 2017; Smith & Paladino, 2010) have stated that having a better knowledge of organic food leads to positive attitudes toward it and being ready to pay a higher price (Díaz et al., 2012). More knowledge can reduce skepticism and increase trust, resulting in better attitudes and a greater inclination to purchase organic food (Janssen & Hamm, 2012; Pieniak et al., 2010; Renko et al., 2011).

The relationship among subjective norms, attitude, and intention to buy organic food confirms previous findings (Irianto, 2015; Smith & Paladino, 2010). However, some studies have shown a lack of influence of subjective norms on consumers' intention to buy organic food (Yadav & Pathak, 2016; Zayed et al., 2022), possibly because buying organic food has not yet become a social norm in some countries (Yadav & Pathak, 2016). The importance of the subjective norm in our conceptual model may reflect the specific context of the organic market in our country. The quality of organic food is contested in public discourse, mainly due to consumers' inadequate awareness and knowledge. As de Maya et al. (2011) stated, many consumers may not have enough information to trust organic products in such circumstances. As a result, consumers may rely more on the opinions of others when deciding whether to purchase organic products. Therefore, the use of opinion leaders, such as celebrities, can be an effective way to encourage people to buy organic food.

The study found that health consciousness influenced the intention to buy organic food directly and indirectly. The effect of health consciousness on attitude (Lee, 2016; Nguyen et al., 2019; Yadav & Pathak, 2016) and intention to buy organic food (Asif et al., 2018; Grubor & Djokic, 2016; Misra & Singh, 2016; Smith & Paladino, 2010; Wang et al., 2019; Yadav & Pathak, 2016) has been reported in other studies, meaning that consumers' beliefs about the safety and health of the product influence their intention to purchase. According to the study of Michaelidou and Hassan (2010), health consciousness was the least significant driver of purchase motivation among the investigated factors. However, in the present study, in line with a recent study (Wang et al., 2019), health consciousness was identified as the most important predictor of the intention to buy organic food. This finding suggests that consumers in the present study emphasize health as a crucial factor when purchasing food products. Therefore, it is important to promote the health benefits of organic food to consumers as these products are often perceived as healthier than non‐organic options (Lea & Worsley, 2005).

The relationship between environmental concern and organic purchase intention validates the findings of previous studies (Pagiaslis & Krontalis, 2014). Research in India (Yadav & Pathak, 2016), Australia (Smith & Paladino, 2010), and America (Lee, 2016) found that environmental concerns positively impacted attitudes but not purchase intent. This may be because these consumers are less inclined to engage in altruistic behavior than their counterparts in other countries (Yadav & Pathak, 2016). In the present study, both environmental concern and health consciousness positively impacted organic food purchasing. The effect coefficients of these variables suggest that Tehrani consumers exhibit a higher inclination toward purchasing organic food for egoistic motives than altruistic ones.

In many developing countries, a lack of access to markets and market information has been identified as an obstacle to consuming organic food (Singh & Verma, 2017). This finding is supported by the present study, showing that the perceived convenience of purchase directly and indirectly influences the intention to purchase organic food. Consumers prefer easily accessible products and do not like to spend too much time searching for organic food (Young et al., 2010). Proper distribution can increase consumer access to organic food, potentially leading to higher consumption rates (Bravo et al., 2013; Melkonyan et al., 2020). However, according to Smith and Paladino's study (2010), organic food accessibility did not affect Australian consumer attitudes, purchase intentions, or behavior. The researchers concluded that accessibility may not significantly affect the purchase of these products in Australia compared to other countries. Nevertheless, they noted that further research is needed to confirm this claim.

The cost difference between organic and non‐organic food has been reported as a major hindrance to purchasing it (Aertsens et al., 2009; Durham & Andrade, 2005; Nguyen et al., 2019). Organic foods are typically more expensive due to their health and environmental benefits (Bravo et al., 2013; Hemmerling et al., 2015). Previous studies have shown that consumers are generally unwilling to pay extra for organic foods (Hossain & Lim, 2016) and often prefer not to buy these products because of their high prices (Akbari & Asadi, 2008). Similarly, the present study found that the intention to purchase organic foods is negatively influenced by perceived price. This issue can be explained by the significant financial constraints of consumers in emerging markets. Therefore, when devising marketing and promotional strategies for organic products, it is vital to consider price and accessibility as crucial parameters.

Our results showed that the product's sensory characteristics, including taste, texture, appearance, and packaging, directly impacted purchase intention. This finding supports previous research that shows that higher‐quality organic food increases consumers' purchase intention without significantly affecting their attitude (Smith & Paladino, 2010). In contrast, the results of studies by Chen (2007) and Lee and Yun (2015) indicated the effect of sensory characteristics of organic products, such as taste, smell, and texture, on consumers' attitudes toward these products. In general, the sensory appeal of organic food is an essential factor influencing purchasing decisions (Massey et al., 2018). Several studies have shown that taste (Aertsens et al., 2011; Sangkumchaliang & Huang, 2012), appearance (Ghorbani & Hamraz, 2009), freshness (Roitner‐Schobesberger et al., 2008; Sangkumchaliang & Huang, 2012), and product packaging (Ibitoye et al., 2014) influence the consumption of organic products. Therefore, it is necessary to consider these things in marketing organic products.

The present study showed that household size and age indirectly positively impacted the purchasing intention of organic food. The education level positively affected the intention to buy these products directly and indirectly. No relationship was found between attitude and intention to buy organic food in men. In women, the intention to buy these products was not affected by environmental concerns or household size. It appears that women prioritize health over environmental concerns when buying organic food. Although sociodemographic characteristics are generally assumed to be significant predictors of organic food consumption, comparing different studies shows conflicting results regarding these relationships' direction and strength. For instance, studies conducted in Thailand (Roitner‐Schobesberger et al., 2008), Germany (Bravo et al., 2013), and Switzerland (Siegrist & Hartmann, 2019), consistent with the findings of the present study, have shown that consumers of organic foods are older and have a university education. The study conducted in America (Durham & Andrade, 2005) reported results contradicting the abovementioned findings. However, according to Li et al. (2007), purchasing organic food is better predicted by attitudinal and behavioral variables rather than demographics.

## 6 CONCLUSION, LIMITATIONS, AND SCOPE FOR FUTURE RESEARCH

The study found that attitude, environmental concern, and sensory characteristics directly affected the intention to purchase organic food. On the other hand, knowledge, perceived price, age, and household size had only an indirect impact. Subjective norms, health consciousness, perceived convenience of purchase, and education level impacted the intention to purchase these products directly and indirectly through attitude. All research variables, except for perceived price, positively affected purchase intention. Health consciousness had the greatest impact, followed by subjective norms and education level. On the other hand, household size, perceived price, and knowledge had the least influence on the intention to buy organic food. In terms of theoretical implications, this study demonstrates that although the power of the TPB model to explain purchase intentions for organic foods is undeniable (Ahmed et al., 2021; Boobalan et al., 2021; Dorce et al., 2021; Fleșeriu et al., 2020; Nguyen et al., 2021; Yang et al., 2018; Yazdanpanah & Forouzani, 2015; Zagata, 2012), the addition of other behavioral variables, particularly knowledge, health consciousness, environmental concern, perceived price, perceived convenience of purchase, and sensory characteristics, enhances our understanding of consumers' behavioral intentions.

These findings could be valuable to a wide range of individuals and groups. For instance, marketers must understand why people choose organic food to develop effective strategies for attracting and retaining loyal customers. Similarly, the packaging industry can benefit from this knowledge by designing appropriate packaging that stimulates consumer behavior and influences purchases. The research also suggests that farmers should switch to organic farming, which benefits the environment and health. Additionally, it can be profitable for them, as it aligns with what consumers want. These findings have implications for policymakers. The government should establish appropriate legislation to support the production and distribution of organic food. Such measures are necessary in order to increase the availability of organic food in the market.

The present study has some limitations. First, the study only examined consumer purchase intentions without assessing their behavior. Although behavioral intention is the most direct predictor of actual behavior (Armitage & Conner, 2001; Sheeran, 2002), future studies can examine how each variable in the model impacts behavior. Second, this study has investigated organic food in general. As the reasons for purchasing and consuming various organic foods can be diverse (McEachern & Willock, 2004), future research could focus on categorizing organic products into groups such as meat, vegetables, dairy products, and so on. According to Padel and Foster (2005), the classification of organic products is a crucial factor in comprehending consumers' decision‐making process and the trade‐offs they encounter. Third, all participants were selected from Tehran, the capital of Iran. So, our findings cannot be generalized to Iran because cultural factors are essential in accepting organic food. Furthermore, the current research may be influenced by self‐selection bias, as the sample may overrepresent individuals who are health conscious, environmentally concerned, and knowledgeable about organic food. Considering the limited sample of Iranian consumers used in the study, it is recommended that the proposed conceptual model be further validated in future studies. This should include different sociodemographic groups, such as those based on age, gender, education level, income, and ethnicity.

## AUTHOR CONTRIBUTIONS

**Marjan Bazhan:** Conceptualization (lead); data curation (lead); formal analysis (lead); funding acquisition (lead); investigation (lead); methodology (lead); project administration (equal); software (equal); supervision (lead); validation (lead); visualization (lead); writing – original draft (equal); writing – review and editing (lead). **Farnam Shafiei Sabet:** Project administration (equal); software (equal); writing – original draft (equal). **Nasrin Borumandnia:** Formal analysis (supporting); methodology (supporting); software (supporting).

## CONFLICT OF INTEREST STATEMENT

The authors state they do not have any competing interests.

## ETHICS STATEMENT

The National Nutrition and Food Technology Research Institute Ethics Committee, Shahid Beheshti University of Medical Sciences, approved the study (IR.SBMU.NNFTRI.REC.1397.240). Participants provided written informed consent before the beginning of the study.

## Data Availability

The datasets used and/or analyzed during the current study are available from the corresponding author upon reasonable request.

## References

[fsn34015-bib-0001] Aertsens, J. , Mondelaers, K. , Verbeke, W. , Buysse, J. , & Van Huylenbroeck, G. (2011). The influence of subjective and objective knowledge on attitude, motivations and consumption of organic food. British Food Journal, 113, 1353–1378. 10.1108/00070701111179988

[fsn34015-bib-0002] Aertsens, J. , Verbeke, W. , Mondelaers, K. , & Van Huylenbroeck, G. (2009). Personal determinants of organic food consumption: A review. British Food Journal, 111, 1140–1167. 10.1108/00070700910992961

[fsn34015-bib-0003] Ahmed, N. , Li, C. , Khan, A. , Qalati, S. A. , Naz, S. , & Rana, F. (2021). Purchase intention toward organic food among young consumers using theory of planned behavior: Role of environmental concerns and environmental awareness. Journal of Environmental Planning and Management, 64, 796–822.

[fsn34015-bib-0004] Ajzen, I. (1991). The theory of planned behavior. Organizational Behavior and Human Decision Processes, 50, 179–211. doi:10.1016/0749-5978(1091)90020-T

[fsn34015-bib-0005] Akbari, M. , & Asadi, A. (2008). A comparative study of Iranian consumers' versus extension experts' attitudes towards agricultural organic products (AOP). Amer. J. Agri. Bio. Sci, 3, 551–558.

[fsn34015-bib-0006] Andervazh, L. , Jalili, S. , & Zanjani, S. (2020). Studying the factors affecting the attitude and intention of buying organic food consumers: Structural equation model. Iranian Journal of Health Education and Health Promotion, 8, 35–44. http://journal.ihepsa.ir/article‐31‐1323‐en.html

[fsn34015-bib-0007] Armitage, C. J. , & Conner, M. (2001). Efficacy of the theory of planned behaviour: A meta‐analytic review. British Journal of Social Psychology, 40, 471–499. 11795063 10.1348/014466601164939

[fsn34015-bib-0008] Arvola, A. , Vassallo, M. , Dean, M. , Lampila, P. , Saba, A. , Lähteenmäki, L. , & Shepherd, R. (2008). Predicting intentions to purchase organic food: The role of affective and moral attitudes in the theory of planned behaviour. Appetite, 50, 443–454. 18036702 10.1016/j.appet.2007.09.010

[fsn34015-bib-0009] Asif, M. , Xuhui, W. , Nasiri, A. , & Ayyub, S. (2018). Determinant factors influencing organic food purchase intention and the moderating role of awareness: A comparative analysis. Food Quality and Preference, 63, 144–150.

[fsn34015-bib-0010] Bagher, A. N. , Salati, F. , & Ghaffari, M. (2018). Factors affecting intention to purchase organic food products among Iranian consumers. Academy of Marketing Studies Journal, 22, 1–23.

[fsn34015-bib-0011] Barnes, A. P. , Vergunst, P. , & Topp, K. (2009). Assessing the consumer perception of the term “organic”: A citizens' jury approach. British Food Journal, 111, 155–164.

[fsn34015-bib-0012] Bazhan, M. , Shafiei Sabet, F. , & Borumandnia, N. (2023). Development and validation of a questionnaire to examine determinants of consumer intentions to purchase organic food. BMC Nutrition, 9, 1–10. 37365648 10.1186/s40795-023-00731-y PMC10291792

[fsn34015-bib-0013] Boobalan, K. , Nawaz, N. , Harindranath, R. M. , & Gajenderan, V. (2021). Influence of altruistic motives on organic food purchase: Theory of planned behavior. Sustainability, 13, 6023.

[fsn34015-bib-0014] Bravo, C. P. , Cordts, A. , Schulze, B. , & Spiller, A. (2013). Assessing determinants of organic food consumption using data from the German National Nutrition Survey II. Food Quality and Preference, 28, 60–70. 10.1016/j.foodqual.2012.1008.1010

[fsn34015-bib-0015] Byrne, P. J. , Toensmeyer, U. C. , German, C. L. , & Muller, H. R. (1991). Analysis of consumer attitudes toward organic produce and purchase likelihood. Journal of Food Distribution Research, 22, 49–62.

[fsn34015-bib-0016] Carfora, V. , Cavallo, C. , Caso, D. , Del Giudice, T. , De Devitiis, B. , Viscecchia, R. , Nardone, G. , & Cicia, G. (2019). Explaining consumer purchase behavior for organic milk: Including trust and green self‐identity within the theory of planned behavior. Food Quality and Preference, 76, 1–9.

[fsn34015-bib-0017] Castro, I. A. , Majmundar, A. , Williams, C. B. , & Baquero, B. (2018). Customer purchase intentions and choice in food retail environments: A scoping review. International Journal of Environmental Research and Public Health, 15, 2493. 30413048 10.3390/ijerph15112493 PMC6266052

[fsn34015-bib-0018] Chakrabarti, S. (2010). Factors influencing organic food purchase in India–expert survey insights. British Food Journal, 112, 902–915.

[fsn34015-bib-0019] Chen, M.‐F. (2007). Consumer attitudes and purchase intentions in relation to organic foods in Taiwan: Moderating effects of food‐related personality traits. Food Quality and Preference, 18, 1008–1021. 10.1016/j.foodqual.2007.1004.1004

[fsn34015-bib-0020] Chen, M.‐F. (2009). Attitude toward organic foods among Taiwanese as related to health consciousness, environmental attitudes, and the mediating effects of a healthy lifestyle. British Food Journal, 111, 165–178. 10.1108/00070700910931986

[fsn34015-bib-0021] Chu, K. M. (2018). Mediating influences of attitude on internal and external factors influencing consumers' intention to purchase organic foods in China. Sustainability, 10, 4690.

[fsn34015-bib-0022] Clark, S. (2020). Organic farming and climate change: The need for innovation. Sustainability, 12, 7012.

[fsn34015-bib-0023] de Maya, S. R. , López‐López, I. , & Munuera, J. L. (2011). Organic food consumption in Europe: International segmentation based on value system differences. Ecological Economics, 70, 1767–1775.

[fsn34015-bib-0024] Díaz, F. J. M. , Pleite, F. M. C. , Paz, J. M. M. , & García, P. G. (2012). Consumer knowledge, consumption, and willingness to pay for organic tomatoes. British Food Journal, 114, 318–334.

[fsn34015-bib-0025] Donald, I. J. , Cooper, S. R. , & Conchie, S. (2014). An extended theory of planned behaviour model of the psychological factors affecting commuters' transport mode use. Journal of Environmental Psychology, 40, 39–48. 10.1016/j.jenvp.2014.1003.1003

[fsn34015-bib-0026] Doni, F. , Zain, C. R. C. M. , Isahak, A. , Fathurrahman, F. , Anhar, A. , Mohamad, W. N.a. W. , Yusoff, W. M. W. , & Uphoff, N. (2018). A simple, efficient, and farmer‐friendly Trichoderma‐based biofertilizer evaluated with the SRI Rice management system. Organic Agriculture, 8, 207–223.

[fsn34015-bib-0027] Dorce, L. C. , da Silva, M. C. , Mauad, J. R. C. , de Faria Domingues, C. H. , & Borges, J. A. R. (2021). Extending the theory of planned behavior to understand consumer purchase behavior for organic vegetables in Brazil: The role of perceived health benefits, perceived sustainability benefits and perceived price. Food Quality and Preference, 91, 104191.

[fsn34015-bib-0028] Dunlap, R. E. , & Jones, R. (2002). Environmental concern: Conceptual & measurement issues. In R. E. Dunlap & W. Michelson (Eds.), Handbook of environmental sociology (pp. 482–542). Greenwood Press.

[fsn34015-bib-0029] Durham, C. A. , & Andrade, D. (2005). Health vs. environmental motivation in organic preferences and purchases, American Agricultural Economics Association Annual Meeting, Providence, RI (July 24–27) .

[fsn34015-bib-0030] Eberle, L. , Milan, G. S. , Borchardt, M. , Pereira, G. M. , & Graciola, A. P. (2022). Determinants and moderators of organic food purchase intention. Food Quality and Preference, 100, 104609.

[fsn34015-bib-0031] Fishbein, M. , & Ajzen, I. (2011). Predicting and changing behavior: The reasoned action approach. Psychology Press.

[fsn34015-bib-0032] Fleșeriu, C. , Cosma, S. A. , & Bocăneț, V. (2020). Values and planned behaviour of the Romanian organic food consumer. Sustainability, 12, 1722.

[fsn34015-bib-0033] Fotopoulos, C. , & Krystallis, A. (2002). Organic product avoidance: Reasons for rejection and potential buyers' identification in a countrywide survey. British Food Journal, 104, 233–260.

[fsn34015-bib-0034] Ghorbani, M. , & Hamraz, S. (2009). A survey on factors affecting on consumer's potential willingness to pay for organic products in Iran (a case study). Trends in Agricultural Economics, 2, 10–16.

[fsn34015-bib-0035] Govindasamy, R. , & Italia, J. (1999). Predicting willingness‐to‐pay a premium for organically grown fresh produce. Journal of Food Distribution Research, 30, 44–53.

[fsn34015-bib-0036] Grubor, A. , & Djokic, N. (2016). Organic food consumer profile in the Republic of Serbia. British Food Journal, 118, 164–182.

[fsn34015-bib-0037] Gundala, R. R. , & Singh, A. (2021). What motivates consumers to buy organic foods? Results of an empirical study in the United States. PLoS One, 16, e0257288. 34506582 10.1371/journal.pone.0257288 PMC8432837

[fsn34015-bib-0038] Gutiérrez‐Villar, B. , Melero‐Bolaños, R. , Montero‐Simo, M. J. , & Araque‐Padilla, R. A. (2022). Profiling consumers with an environmentally sustainable and healthy diet: The case of Spanish households. Frontiers in Nutrition, 9, 1035142. 36438776 10.3389/fnut.2022.1035142 PMC9684671

[fsn34015-bib-0039] Guzmán, G. I. , & Alonso, A. M. (2008). A comparison of energy use in conventional and organic olive oil production in Spain. Agricultural Systems, 98, 167–176.

[fsn34015-bib-0040] Hemmerling, S. , Hamm, U. , & Spiller, A. (2015). Consumption behaviour regarding organic food from a marketing perspective—A literature review. Organic Agriculture, 5, 277–313.

[fsn34015-bib-0041] Hooman, H. (2016). Structural equation modeling using Lisrel application. Samt [in Persian].

[fsn34015-bib-0042] Hossain, M. , & Lim, P. (2016). Consumers' buying behavior towards organic foods: Evidence from the emerging market. Malaysian Management Review, 51, 7–25.

[fsn34015-bib-0043] Hosseini, N. M. (2019). An overview of the organic farming situation in Iran (Challenges and Solutions) . ACTA Scientific Agriculture (ISSN: 2581‐365X), 3.1183‐1187.

[fsn34015-bib-0044] Huber, M. , Bakker, M. H. , Dijk, W. , Prins, H. A. , & Wiegant, F. A. (2012). The challenge of evaluating health effects of organic food; operationalisation of a dynamic concept of health. Journal of the Science of Food and Agriculture, 92, 2766–2773. 22252459 10.1002/jsfa.5563

[fsn34015-bib-0045] Ibitoye, O. , Nawi, N. M. , Kamarulzaman, N. H. , & Man, N. (2014). Consumers' awareness towards organic rice in Malaysia. International Food Research Journal, 21, 1711–1718.

[fsn34015-bib-0046] Imani, B. , Allahyari, M. S. , Bondori, A. , Surujlal, J. , & Sawicka, B. (2021). Determinants of organic food purchases intention: The application of an extended theory of planned behaviour .

[fsn34015-bib-0047] Irianto, H. (2015). Consumers' attitude and intention towards organic food purchase: An extension of theory of planned behavior in gender perspective. International Journal of Management, Economics and Social Sciences, 4, 17–31.

[fsn34015-bib-0048] Iversen, A. C. , & Kraft, P. (2006). Does socio‐economic status and health consciousness influence how women respond to health related messages in media? Health Education Research, 21, 601–610. 16702193 10.1093/her/cyl014

[fsn34015-bib-0049] Janssen, M. (2018). Determinants of organic food purchases: Evidence from household panel data. Food Quality and Preference, 68, 19–28.

[fsn34015-bib-0050] Janssen, M. , & Hamm, U. (2012). Product labelling in the market for organic food: Consumer preferences and willingness‐to‐pay for different organic certification logos. Food Quality and Preference, 25, 9–22.

[fsn34015-bib-0051] Khosh‐Khui, M. (2016). A General Review of Organic Agriculture Strategic Research. Journal of Agricultural Sciences and Natural Resources, 1, 35–50. [In persian].

[fsn34015-bib-0052] Kline, R. B. (2011). Principles and practice of structural equation modeling (3rd ed.). The Guildford Press.

[fsn34015-bib-0053] Lea, E. , & Worsley, T. (2005). Australians' organic food beliefs, demographics and values. British Food Journal, 107, 855–869.

[fsn34015-bib-0054] Lee, H.‐J. (2016). Individual and situational determinants of US consumers' buying behavior of organic foods. Journal of International Food & Agribusiness Marketing, 28, 117–131. 10.1080/08974438.08972015.01035471

[fsn34015-bib-0055] Lee, H.‐J. , & Yun, Z.‐S. (2015). Consumers' perceptions of organic food attributes and cognitive and affective attitudes as determinants of their purchase intentions toward organic food. Food Quality and Preference, 39, 259–267.

[fsn34015-bib-0056] Li, J. , Zepeda, L. , & Gould, B. W. (2007). The demand for organic food in the US: An empirical assessment. Journal of Food Distribution Research, 38, 54–69.

[fsn34015-bib-0057] Liobikienė, G. , Mandravickaitė, J. , & Bernatonienė, J. (2016). Theory of planned behavior approach to understand the green purchasing behavior in the EU: A cross‐cultural study. Ecological Economics, 125, 38–46.

[fsn34015-bib-0058] Lockie, S. , Lyons, K. , Lawrence, G. , & Grice, J. (2004). Choosing organics: A path analysis of factors underlying the selection of organic food among Australian consumers. Appetite, 43, 135–146. 15458800 10.1016/j.appet.2004.02.004

[fsn34015-bib-0059] Loebnitz, N. , & Aschemann‐Witzel, J. (2016). Communicating organic food quality in China: Consumer perceptions of organic products and the effect of environmental value priming. Food Quality and Preference, 50, 102–108.

[fsn34015-bib-0060] MacKinnon, D. P. , Lockwood, C. M. , & Williams, J. (2004). Confidence limits for the indirect effect: Distribution of the product and resampling methods. Multivariate Behavioral Research, 39, 99–128. 20157642 10.1207/s15327906mbr3901_4 PMC2821115

[fsn34015-bib-0061] Magnusson, M. K. , Arvola, A. , Koivisto Hursti, U.‐K. , Åberg, L. , & Sjödén, P.‐O. (2001). Attitudes towards organic foods among Swedish consumers. British Food Journal, 103, 209–227.

[fsn34015-bib-0062] Massey, M. , O'Cass, A. , & Otahal, P. (2018). A meta‐analytic study of the factors driving the purchase of organic food. Appetite, 125, 418–427. 29501680 10.1016/j.appet.2018.02.029

[fsn34015-bib-0063] McEachern, M. G. , & Willock, J. (2004). Producers and consumers of organic meat: A focus on attitudes and motivations. British Food Journal, 106, 534–552. 10.1108/00070700410545737

[fsn34015-bib-0064] Melkonyan, A. , Gruchmann, T. , Lohmar, F. , Kamath, V. , & Spinler, S. (2020). Sustainability assessment of last‐mile logistics and distribution strategies: The case of local food networks. International Journal of Production Economics, 228, 107746.

[fsn34015-bib-0065] Michaelidou, N. , & Hassan, L. M. (2010). Modeling the factors affecting rural consumers' purchase of organic and free‐range produce: A case study of consumers' from the Island of arran in Scotland, UK. Food Policy, 35, 130–139. 10.1016/j.foodpol.2009.1010.1001

[fsn34015-bib-0066] Misra, R. , & Singh, D. (2016). An analysis of factors affecting growth of organic food. British Food Journal, 118, 2308–2325.

[fsn34015-bib-0067] Moser, A. K. (2016). Buying organic–decision‐making heuristics and empirical evidence from Germany. Journal of Consumer Marketing, 33, 552–561.

[fsn34015-bib-0068] Nguyen, H. V. , Nguyen, N. , Nguyen, B. K. , & Greenland, S. (2021). Sustainable food consumption: Investigating organic meat purchase intention by Vietnamese consumers. Sustainability, 13, 953.

[fsn34015-bib-0069] Nguyen, H. V. , Nguyen, N. , Nguyen, B. K. , Lobo, A. , & Vu, P. A. (2019). Organic food purchases in an emerging market: The influence of consumers' personal factors and green marketing practices of food stores. International Journal of Environmental Research and Public Health, 16, 1037. 10.3390/ijerph16061037 30909390 PMC6466195

[fsn34015-bib-0070] Onwezen, M. C. , Antonides, G. , & Bartels, J. (2013). The norm activation model: An exploration of the functions of anticipated pride and guilt in pro‐environmental behaviour. Journal of Economic Psychology, 39, 141–153.

[fsn34015-bib-0071] Padel, S. , & Foster, C. (2005). Exploring the gap between attitudes and behaviour: Understanding why consumers buy or do not buy organic food. British Food Journal, 107, 606–625. 10.1108/00070700510611002

[fsn34015-bib-0072] Pagiaslis, A. , & Krontalis, A. K. (2014). Green consumption behavior antecedents: Environmental concern, knowledge, and beliefs. Psychology & Marketing, 31, 335–348. 10.1002/mar.20698

[fsn34015-bib-0073] Parashar, S. , Singh, S. , & Sood, G. (2023). Examining the role of health consciousness, environmental awareness and intention on purchase of organic food: A moderated model of attitude. Journal of Cleaner Production, 386, 135553.

[fsn34015-bib-0074] Paul, J. , & Rana, J. (2012). Consumer behavior and purchase intention for organic food. Journal of Consumer Marketing, 29, 412–422.

[fsn34015-bib-0075] Pieniak, Z. , Aertsens, J. , & Verbeke, W. (2010). Subjective and objective knowledge as determinants of organic vegetables consumption. Food Quality and Preference, 21, 581–588.

[fsn34015-bib-0076] Rana, J. , & Paul, J. (2017). Consumer behavior and purchase intention for organic food: A review and research agenda. Journal of Retailing and Consumer Services, 38, 157–165.

[fsn34015-bib-0077] Rembiałkowska, E. (2007). Quality of plant products from organic agriculture. Journal of the Science of Food and Agriculture, 87, 2757–2762. 10.1002/jsfa.3000

[fsn34015-bib-0078] Renko, S. , Vignali, C. , & Żakowska‐Biemans, S. (2011). Polish consumer food choices and beliefs about organic food. British Food Journal, 113, 122–137.

[fsn34015-bib-0079] Rimal, A. P. , Moon, W. , & Balasubramanian, S. (2005). Agro‐biotechnology and organic food purchase in the United Kingdom. British Food Journal, 107, 84–97. 10.1108/00070700510579162

[fsn34015-bib-0080] Roitner‐Schobesberger, B. , Darnhofer, I. , Somsook, S. , & Vogl, C. R. (2008). Consumer perceptions of organic foods in Bangkok, Thailand. Food Policy, 33, 112–121. 10.1016/j.foodpol.2007.1009.1004

[fsn34015-bib-0081] Sadiq, W. , Abdullah, I. , Aslam, K. , & Zulfiqar, S. (2020). Engagement marketing: The innovative perspective to enhance the viewer's loyalty in social media and blogging e‐commerce websites. Marketing & Management of Innovations, 1, 149–166.

[fsn34015-bib-0082] Sangkumchaliang, P. , & Huang, W.‐C. (2012). Consumers' perceptions and attitudes of organic food products in northern Thailand. International Food and Agribusiness Management Review, 15, 87–102. 10.22004/ag.econ.120860

[fsn34015-bib-0083] Scialabba, N. E.‐H. , & Müller‐Lindenlauf, M. (2010). Organic agriculture and climate change. Renewable Agriculture and Food Systems, 25, 158–169.

[fsn34015-bib-0084] Sheeran, P. (2002). Intention—Behavior relations: A conceptual and empirical review. European Review of Social Psychology, 12, 1–36.

[fsn34015-bib-0085] Siegrist, M. , & Hartmann, C. (2019). Impact of sustainability perception on consumption of organic meat and meat substitutes. Appetite, 132, 196–202. 10.1016/j.appet.2018.1009.1016 30322657

[fsn34015-bib-0086] Singh, A. , & Verma, P. (2017). Factors influencing Indian consumers' actual buying behaviour towards organic food products. Journal of Cleaner Production, 167, 473–483. 10.1016/j.jclepro.2017.1008.1106

[fsn34015-bib-0087] Smith, O. M. , Cohen, A. L. , Rieser, C. J. , Davis, A. G. , Taylor, J. M. , Adesanya, A. W. , Jones, M. S. , Meier, A. R. , Reganold, J. P. , & Orpet, R. J. (2019). Organic farming provides reliable environmental benefits but increases variability in crop yields: A global meta‐analysis. Frontiers in Sustainable Food Systems, 3, 82.

[fsn34015-bib-0088] Smith, S. , & Paladino, A. (2010). Eating clean and green? Investigating consumer motivations towards the purchase of organic food. Australasian Marketing Journal; AMJ, 18, 93–104. 10.1016/j.ausmj.2010.1001.1001

[fsn34015-bib-0089] Squires, L. , Juric, B. , & Bettina Cornwell, T. (2001). Level of market development and intensity of organic food consumption: Cross‐cultural study of Danish and New Zealand consumers. Journal of Consumer Marketing, 18, 392–409.

[fsn34015-bib-0090] Stanton, J. V. , & Cook, L. A. (2019). Product knowledge and information processing of organic foods. Journal of Consumer Marketing, 36, 240–252.

[fsn34015-bib-0091] Steptoe, A. , Pollard, T. M. , & Wardle, J. (1995). Development of a measure of the motives underlying the selection of food: The food choice questionnaire. Appetite, 25, 267–284. https://www.ucl.ac.uk/hbrc/diet/SteptoeandWardleFCQ.pdf 8746966 10.1006/appe.1995.0061

[fsn34015-bib-0092] Tabachnick, B. G. , Fidell, L. S. , & Ullman, J. B. (2007). Using multivariate statistics. Pearson.

[fsn34015-bib-0093] Tarkiainen, A. , & Sundqvist, S. (2005). Subjective norms, attitudes and intentions of Finnish consumers in buying organic food. British Food Journal, 107, 808–822. 10.1108/00070700510629760

[fsn34015-bib-0094] Thøgersen, J. (2016). Consumer decision‐making with regard to organic food products, traditional food production and rural sustainable development (pp. 173–192). Routledge.

[fsn34015-bib-0095] Timu, A. , Gustafson, C. , & Azzam, A. (2022). Measuring organic versus conventional food preferences: The case of fruits and vegetables . Available at SSRN 4032733.

[fsn34015-bib-0096] Tran, A. T. V. , & Nguyen, N. T. (2021). Organic food consumption among households in Hanoi: Importance of situational factors. Sustainability, 13, 12496.

[fsn34015-bib-0097] Vilnai‐Yavetz, I. , & Koren, R. (2013). Cutting through the clutter: Purchase intentions as a function of packaging instrumentality, aesthetics, and symbolism. The International Review of Retail, Distribution and Consumer Research, 23, 394–417.

[fsn34015-bib-0098] Wang, X. , Pacho, F. , Liu, J. , & Kajungiro, R. (2019). Factors influencing organic food purchase intention in developing countries and the moderating role of knowledge. Sustainability, 11, 209. 10.3390/su11010209

[fsn34015-bib-0099] Willer, H. , & Sahota, A. (2020). The world of organic agriculture, statistics and emerging trends 2020 at BIOFACH 2020, BIOFACH congress 2020. Messezentrum Nürnberg.

[fsn34015-bib-0100] Willer, H. , Schlatter, B. , & Trávníček, J. (2023). The world of organic agriculture. Statistics and emerging trends 2023. Research Institute of Organic Agriculture FiBL, Frick, and IFOAM–organics international.

[fsn34015-bib-0101] Yadav, R. , & Pathak, G. S. (2016). Intention to purchase organic food among young consumers: Evidences from a developing nation. Appetite, 96, 122–128. 10.1016/j.appet.2015.1009.1017 26386300

[fsn34015-bib-0102] Yang, S. , Li, L. , & Zhang, J. (2018). Understanding consumers' sustainable consumption intention at China's double‐11 online shopping festival: An extended theory of planned behavior model. Sustainability, 10, 1801.

[fsn34015-bib-0103] Yazdanpanah, M. , & Forouzani, M. (2015). Application of the theory of planned behaviour to predict Iranian students' intention to purchase organic food. Journal of Cleaner Production, 107, 342–352.

[fsn34015-bib-0104] Yazdanpanah, M. , Forouzani, M. , & Hojjati, M. (2015). Willingness of Iranian young adults to eat organic foods: Application of the health belief model. Food Quality and Preference, 41, 75–83. 10.1016/j.foodqual.2014.1011.1012

[fsn34015-bib-0105] Yeganeh, H. , Farsi, R. , Frozeh, M. R. , Pournemati, A. , & Mirdeilami, S. Z. (2022). A study of variables affecting the consumption of medicinal plant products in urban communities, north of Iran. Environment, Development and Sustainability, 24, 7455–7469.

[fsn34015-bib-0106] Young, W. , Hwang, K. , McDonald, S. , & Oates, C. J. (2010). Sustainable consumption: Green consumer behaviour when purchasing products. Sustainable Development, 18, 20–31.

[fsn34015-bib-0107] Yu, J. , & Wu, J. (2018). The sustainability of agricultural development in China: The agriculture–environment nexus. Sustainability, 10, 1776.

[fsn34015-bib-0108] Zagata, L. (2012). Consumers' beliefs and behavioural intentions towards organic food. Evidence from the Czech Republic. Appetite, 59, 81–89. 22504401 10.1016/j.appet.2012.03.023

[fsn34015-bib-0109] Zanoli, R. , & Naspetti, S. (2002). Consumer motivations in the purchase of organic food: A means‐end approach. British Food Journal, 104, 643–653. 10.1108/00070700210425930

[fsn34015-bib-0110] Zayed, M. F. , Gaber, H. R. , & El Essawi, N. (2022). Examining the factors that affect consumers' purchase intention of organic food products in a developing country. Sustainability, 14, 5868.

